# National Prevalence of Excessive Screen Exposure Among Chinese Preschoolers

**DOI:** 10.1001/jamanetworkopen.2022.24244

**Published:** 2022-07-28

**Authors:** Jing Hua, Jinhong Xie, Charlie Baker, Wenchong Du

**Affiliations:** 1 Department of Mother and Children’s Health Care, Shanghai First Maternity and Infant Hospital, School of Medicine, Tongji University, Shanghai, China; 2 NTU Psychology, Nottingham Trent University, Nottingham, United Kingdom

## Abstract

This cohort study uses data from the Chinese National Cohort of Motor Development to evaluate the national prevalence of and factors associated with excessive screen exposure among Chinese preschoolers aged 3 to 5 years.

## Introduction

Excessive screen exposure among children has been associated with negative outcomes in neurodevelopment, learning, memory, and mental health,^1^ and the increase in screen exposure, including television and digital devices, among children has become a global public health issue. However, to our knowledge, no previous studies have evaluated the national prevalence of excessive screen exposure among Chinese children. This study aimed to evaluate the national prevalence of and factors associated with excessive screen exposure among Chinese preschoolers aged 3 to 5 years in a population-representative sample.

## Methods

Data for this retrospective cohort study, conducted from April 1, 2018, to December 31, 2019, were obtained from the Chinese National Cohort of Motor Development, with data collected from 551 locations in China.^2^ A total of 129 278 children with complete information on personal characteristics (Table 1) were included in the final analysis. The study was approved by the Ethics Committee of Shanghai First Maternity and Infant Hospital. All information acquired was kept confidential and was accessible only by the researchers. Parents provided online written consent to participate in the study before completing the online questionnaire. This study followed the Strengthening the Reporting of Observational Studies in Epidemiology (STROBE) reporting guideline for cohort studies.

**Table 1.** Daily Screen Exposure Hours by Individual and Family Characteristics in the Study Population

Characteristic	No. (%) (N = 129 278)	Screen time during the weekday, mean (SD), h	*P* value	Screen time during the weekend, mean (SD), h	*P* value
Child’s age, y					
3	43 661 (33.8)	1.3 (1.0)	<.001	2.5 (1.8)	<.001
4	48 531 (37.5)	1.3 (1.0)		2.6 (1.9)	
5	37 086 (28.7)	1.3 (0.9)		2.7 (1.9)	
Sex					
Male	67 780 (52.4)	1.3 (1.0)	<.001	2.7 (1.9)	<.001
Female	61 498 (47.6)	1.2 (0.9)		2.5 (1.8)	
Child’s BMI					
≤18	119 030 (92.1)	1.3 (0.9)	<.001	2.6 (1.8)	<.001
>18	10 248 (7.9)	1.4 (1.0)		2.9 (2.0)	
Physical activity, min					
≥180	65 008 (50.3)	1.3 (1.0)	<.001	2.7 (1.9)	<.001
<180	64 270 (49.7)	1.2 (0.9)		2.5 (1.8)	
Maternal age at delivery, y					
<30	95 915 (74.2)	1.3 (1.0)	<.001	2.6 (1.9)	<.001
30-34	25 007 (19.3)	1.2 (0.9)		2.4 (1.8)	
≥35	8356 (6.5)	1.2 (0.9)		2.5 (1.8)	
Higher education of mother					
No	58 862 (45.5)	1.1 (0.9)	<.001	2.2 (1.7)	<.001
Yes	70 416 (54.5)	1.5 (1.0)		3.1 (1.0)	
Higher education of father					
No	60 013 (46.4)	1.1 (0.9)	<.001	2.2 (1.7)	<.001
Yes	69 265 (53.6)	1.5 (1.0)		3.0 (2.0)	
Mother’s occupation					
Employed	107 606 (83.2)	1.3 (0.9)	<.001	2.5 (1.8)	<.001
Unemployed	21 672 (16.8)	1.4 (1.0)		2.8 (1.9)	
Father’s occupation					
Employed	125 431 (97.0)	1.3 (0.9)	<.001	2.6 (1.8)	<.001
Unemployed	3847 (3.0)	1.5 (1.1)		3.0 (2.1)	
Family annual per-capita income, ¥^a^					
Below national mean income	32 851 (25.4)	1.4 (1.0)	<.001	2.7 (1.9)	<.001
Above or equal to national mean income	96 427 (74.6)	1.3 (0.9)		2.5 (1.8)	
Family structure					
Single-parent families	3200 (2.5)	1.4 (1.0)	<.001	3.0 (2.0)	<.001
Nuclear families with both parents	79 952 (61.8)	1.3 (1.0)		2.6 (1.9)	
Extended families with grandparents	46 126 (35.7)	1.3 (0.9)		2.6 (1.8)	
No. of children in the family					
1	58 019 (44.9)	1.3 (1.0)	<.001	2.8 (1.9)	<.001
≥2	71 259 (55.1)	1.2 (0.9)		2.5 (1.9)	

Abbreviation: BMI, body mass index (calculated as weight in kilograms divided by height in meters squared).

^a^ National mean family per-capita income in the year before the survey.

Parents reported children’s daily screen exposure time based on the child’s typical time spent in the past year watching television or using a smartphone, a computer, or a tablet on weekdays and weekends (eTable in the Supplement). Daily screen exposure time was calculated as the value of 5/7 × screen exposure hours during weekdays + 2/7 × screen exposure hours during weekends. Excessive screen exposure was defined as daily screen exposure time exceeding 1 and 2 hours according to the World Health Organization^3^ and the American Association of Pediatrics^4,5^ guidelines, respectively. Information on sociodemographic characteristics (child’s age and sex, parental educational level, family income, parental employment, mother’s age, family structure, and sibling status) and child’s body mass index (BMI) and physical activity level wase collected from the parents.

Statistical analysis was conducted from April 1, 2019, to December 31, 2022. Descriptive statistics procedures (frequency distribution and mean [SD] values) were used. One-way analysis of variance was conducted to compare the screen exposure time among preschoolers with different personal characteristics. Multivariable logistic regression analysis was conducted to investigate the associations of family and child characteristics with the prevalence of excessive screen exposure. Analyses were performed using SPSS, version 20 (SPSS Inc). All *P* values were from 2-sided tests and results were deemed statistically significant at *P* < .05.

## Results

This study included 129 278 children (67 780 boys [52.4%]; mean [SD] age, 4.0 [0.8] years) (Table 1). The mean (SD) weekday daily screen exposure time was 1.3 (1.0) hours, and the mean (SD) weekend daily screen exposure time was 2.6 (1.9) hours. A total of 86 728 children (67.1%) had more than 1 hour of daily screen exposure time, and 37 362 children (28.9%) had more than 2 hours of daily screen exposure time. Overall, daily screen exposure time varied according to different child and family characteristics. Associations were found between child and family characteristics and the prevalence of excessive screen exposure. Compared with girls, boys had greater odds of excessive screen exposure (>1 hour: adjusted odds ratio [aOR], 1.11 [95% CI, 1.08-1.14]; *P* < .001; >2 hours: aOR, 1.15 [95% CI, 1.13-1.18; *P* < .001) (Table 2). Compared with children aged 3 years, those aged 4 years had greater odds of more than 1 hour of excessive screen exposure (aOR, 1.03 [95% CI, 1.00-1.06]; *P* = .04). Children with a BMI (calculated as weight in kilograms divided by height in meters squared) of greater than 18 had greater odds of excessive screen exposure than children with a BMI of 18 or less (>1 hour: aOR, 1.28 [95% CI, 1.22-1.34]; *P* < .001; >2 hours: aOR, 1.24 [95% CI, 1.18-1.29; *P* < .001).

**Table 2.** Association of Child and Family Characteristics With the Prevalence of Excessive Screen Exposure

Characteristic	Excessive screen exposure (>1 h), No. (%) (N = 129 278)		Odds ratio (95% CI)		Excessive screen exposure (>2 h), No. (%) (N = 129 278)		Odds ratio (95% CI)	
	Yes	No	Crude^a^	Adjusted^b^	Yes	No	Crude^a^	Adjusted^b^
Child’s age, y								
3	28 833 (66.0)	14 828 (34.0)	1 [Reference]	1 [Reference]	12 481 (28.6)	31 180 (71.4)	1 [Reference]	1 [Reference]
4	32 543 (67.1)	15 988 (32.9)	1.05 (1.02-1.08)^c^	1.03 (1.00-1.06)^d^	14 067 (29.0)	34 464 (71.0)	1.02 (0.99-1.05)	1.01 (0.98-1.04)
5	25 352 (68.4)	11 734 (31.6)	1.11 (1.08-1.14)^e^	1.02 (0.99-1.06)	10 814 (29.2)	26 272 (70.8)	1.03 (1.00-1.06)	0.96 (0.93-0.99)^d^
Sex								
Female	40 478 (65.8)	21 020 (34.3)	1 [Reference]	1 [Reference]	16 795 (27.3)	44 703 (72.7)	1 [Reference]	1 [Reference]
Male	46 250 (68.2)	21 530 (31.8)	1.12 (1.09-1.14)^e^	1.11 (1.08-1.14)^e^	20 567 (30.3)	47 213 (69.7)	1.16 (1.13-1.18)^e^	1.15 (1.13-1.18)^e^
Child’s body mass index^f^								
≤18	79 246 (66.6)	39 784 (33.4)	1 [Reference]	1 [Reference]	33 850 (28.4)	85 171 (71.6)	1 [Reference]	1 [Reference]
>18	7482 (73.0)	2766 (27.0)	1.36 (1.30-1.42)^e^	1.28 (1.22-1.34)^e^	3503 (34.2)	6745 (65.8)	1.31 (1.25-1.36)^e^	1.24 (1.18-1.29)^e^
Physical activity, min								
≥180	44 842 (69.0)	20 166 (31.0)	1 [Reference]	1 [Reference]	20 796 (32.0)	44 212 (68.0)	1 [Reference]	1 [Reference]
<180	41 886 (65.2)	22 384 (34.8)	0.84 (0.82-0.86)^e^	0.83 (0.81-0.85)^e^	16 566 (25.8)	47 704 (74.2)	0.74 (0.72-0.76)^e^	0.73 (0.71-0.75)^e^
Maternal age at delivery, y								
<30	65 595 (68.4)	30 320 (31.6)	1 [Reference]	1 [Reference]	28 748 (30.0)	67 167 (70.0)	1 [Reference]	1 [Reference]
30-34	15 636 (62.5)	9371 (37.5)	0.78 (0.76-0.81)^e^	0.78 (0.76-0.81)^e^	6326 (25.3)	18 681 (74.7)	0.79 (0.77-0.82)^e^	0.81 (0.78-0.83)^e^
≥35	5497 (65.8)	2859 (34.2)	0.74 (0.70-0.78)^e^	0.74 (0.70-0.78)^e^	2288 (27.4)	6068 (72.6)	0.88 (0.84-0.93)^e^	0.76 (0.72-0.80)^e^
Higher education of mother								
Yes	40 478 (57.5)	29 938 (42.5)	1 [Reference]	1 [Reference]	22 294 (37.9)	36 568 (62.1)	1 [Reference]	1 [Reference]
No	46 250 (78.6)	12 612 (21.4)	2.71 (2.65-2.78)^e^	1.91 (1.84-1.97)^e^	15 068 (21.4)	55 348 (78.6)	2.24 (2.19-2.30)	1.64 (1.59-1.70)
Higher education of father								
Yes	39 969 (57.7)	29 296 (42.3)	1 [Reference]	1 [Reference]	22 574 (37.6)	37 439 (62.4)	1 [Reference]	1 [Reference]
No	46 759 (77.9)	13 254 (22.1)	2.59 (2.52-2.65)^e^	1.67 (1.62-1.73)^e^	14 788 (21.3)	54 477 (78.7)	2.22 (2.17-2.28)^e^	1.59 (1.54-1.64)^e^
Mother’s occupation								
Employed	70 986 (66.0)	36 620 (34.0)	1 [Reference]	1 [Reference]	30 164 (28.0)	77 442 (72.0)	1 [Reference]	1 [Reference]
Unemployed	15 742 (72.6)	5930 (27.5)	1.37 (1.33-1.42)^e^	1.00 (0.96-1.03)	7198 (33.2)	14 474 (66.8)	1.23 (1.24-1.32)^e^	1.01 (0.98-1.04)
Father’s occupation								
Employed	83 768 (66.8)	41 663 (33.2)	1 [Reference]	1 [Reference]	35 992 (28.7)	89 439 (71.3)	1 [Reference]	1 [Reference]
Unemployed	2960 (76.9)	887 (23.1)	1.66 (1.54-1.79)^e^	1.17 (1.08-1.27)^e^	1370 (35.6)	2477 (64.4)	1.37 (1.26-1.47)^e^	1.04 (0.97-1.12)
Family annual per-capita income, ¥^g^								
Above or equal to national mean income	63 663 (66.0)	32 764 (34.0)	1 [Reference]	1 [Reference]	26 967 (28.0)	69 460 (72.0)	1 [Reference]	1 [Reference]
Below national mean income	23 065 (70.2)	9786 (29.8)	1.21 (1.18-1.25)^e^	1.00 (0.96-1.02)	10 395 (31.6)	22 456 (68.4)	1.19 (1.16-1.23)^e^	1.01 (0.98-1.04)
Family structure								
Nuclear families with both parents	53 668 (67.1)	26 284 (32.9)	1 [Reference]	1 [Reference]	22 782 (28.5)	57 170 (71.5)	1 [Reference]	1 [Reference]
Extended families with grandparents	30 663 (66.5)	15 463 (33.5)	0.91 (0.88-0.93)^e^	0.91 (0.88-0.93)^e^	13 416 (29.1)	32 710 (70.9)	0.97 (0.95-1.00)^e^	0.87 (0.85-0.89)^e^
Single-parent families	2397 (74.9)	803 (25.1)	1.03 (1.01-1.06)^d^	1.23 (1.13-1.34)^e^	1164 (36.4)	2036 (63.6)	1.39 (1.29-1.50)^e^	1.18 (1.09-1.28)^e^
No. of children in family								
≥2	45 593 (64.0)	25 666 (36.0)	1 [Reference]	1 [Reference]	18 922 (26.6)	52 337 (73.4)	1 [Reference]	1 [Reference]
1	41 135 (70.9)	16 884 (29.1)	1.37 (1.34-1.40)^e^	1.19 (1.16-1.22)^e^	18 440 (31.8)	39 579 (68.2)	1.29 (1.26-1.32)^e^	1.15 (1.12-1.18)^e^

^a^ Not adjusting for any variables.

^b^ Adjusting for other child and family characteristics.

^c^ *P* < .01.

^d^ *P* < .05.

^e^ *P* < .001.

^f^ Calculated as weight in kilograms divided by height in meters squared.

^g^ National mean family per-capita income in the year before the survey.

## Discussion

To our knowledge, this is the first nationally representative work to report the prevalence of excessive screen exposure among Chinese children. We found a high prevalence of excessive screen exposure among Chinese preschoolers. Excessive screen exposure was associated with male sex, older age, higher BMI, higher physical activity levels, younger mothers, lower family socioeconomic status levels, and 1-child and single-parent status. Living with grandparents was a protective factor against excessive screen exposure. We also found greater weekend daily screen exposure time compared with weekday daily screen exposure time, which may suggest increased screen exposure during the daytime and delayed sleeping time during weekends.

The limitations of this study included potential parent-reported errors and the cross-sectional nature of the data, which does not allow for the determination of causation but only association. Further efforts to understand the determinants of the variations in screen exposure are required. It is necessary to develop interventions to reduce excessive screen time among Chinese preschoolers, particularly for subgroups with a higher prevalence of screen exposure.

## Notes

Supplement. **eTable.** Survey Questions Asking About the Screen Exposure of the Child
