# Supplementary material for: National Prevalence of Excessive Screen Exposure Among Chinese Preschoolers
Source: JAMA Netw Open. 2022 Jul 28;5(7):e2224244. doi: 10.1001/jamanetworkopen.2022.24244 (PMC9335133; doi:10.1001/jamanetworkopen.2022.24244)
Supplement: Supplement. — eTable. Survey Questions Asking About the Screen Exposure of the Child [file jamanetwopen-e2224244-s001.pdf]

Supplementary Online Content

Hua J, Xie J, Baker C, Du W. National prevalence of excessive screen exposure among Chinese preschoolers. *JAMA Netw Open.* 2022;5(7):e2224244. doi:10.1001/jamanetworkopen.2022.24244

eTable. Survey Questions Asking About the Screen Exposure of the Child

This supplementary material has been provided by the authors to give readers additional information about their work.

© 2022 Hua J et al. *JAMA Network Open.*

eTable. Survey Questions Asking About the Screen Exposure of the Child

	Questions to the parents	Answer
1	Consider the typical situation in the past whole year, how many minutes on a weekday does your child usually spend watching TV, using a smartphone, a computer, or a tablet?	
2	Consider the typical situation in the past whole year, how many minutes on a weekend day does your child usually spend watching TV, using a smartphone, a computer, or a tablet?	

© 2022 Hua J et al. *JAMA Network Open.*
